# GlioMarker: An integrated database for knowledge exploration of diagnostic biomarkers in gliomas

**DOI:** 10.3389/fonc.2022.792055

**Published:** 2022-08-11

**Authors:** Zihan Ran, Jingcheng Yang, Yaqing Liu, XiuWen Chen, Zijing Ma, Shaobo Wu, Yechao Huang, Yueqiang Song, Yu Gu, Shuo Zhao, Mengqi Fa, Jiangjie Lu, Qingwang Chen, Zehui Cao, Xiaofei Li, Shanyue Sun, Tao Yang

**Affiliations:** ^1^ Department of Research, Shanghai University of Medicine & Health Sciences Affiliated Zhoupu Hospital, Shanghai, China; ^2^ Inspection and Quarantine Department, The College of Medical Technology, Shanghai University of Medicine & Health Sciences, Shanghai, China; ^3^ The Genius Medicine Consortium (TGMC), Shanghai, China; ^4^ State Key Laboratory of Genetic Engineering, Human Phenome Institute, School of Life Sciences and Shanghai Cancer Center, Fudan University, Shanghai, China; ^5^ Center for Intelligent Medicine Research, Greater Bay Area Institute of Precision Medicine, Guangzhou, China; ^6^ Department of Laboratory Medicine, Tinglin Hospital of Jinshan District, Shanghai, China; ^7^ Department of Toxicology, School of Public Health, Guangxi Medical University, Nanning, China; ^8^ Department of Radiology, Shanghai University of Medicine & Health Sciences Affiliated Zhoupu Hospital, Shanghai, China

**Keywords:** gliomas, biomarker, diagnostic, database, knowledge exploration

## Abstract

Gliomas are the most frequent malignant and aggressive tumors in the central nervous system. Early and effective diagnosis of glioma using diagnostic biomarkers can prolong patients’ lives and aid in the development of new personalized treatments. Therefore, a thorough and comprehensive understanding of the diagnostic biomarkers in gliomas is of great significance. To this end, we developed the integrated and web-based database GlioMarker (http://gliomarker.prophetdb.org/), the first comprehensive database for knowledge exploration of glioma diagnostic biomarkers. In GlioMarker, accurate information on 406 glioma diagnostic biomarkers from 1559 publications was manually extracted, including biomarker descriptions, clinical information, associated literature, experimental records, associated diseases, statistical indicators, etc. Importantly, we integrated many external resources to provide clinicians and researchers with the capability to further explore knowledge on these diagnostic biomarkers based on three aspects. (1) Obtain more ontology annotations of the biomarker. (2) Identify the relationship between any two or more components of diseases, drugs, genes, and variants to explore the knowledge related to precision medicine. (3) Explore the clinical application value of a specific diagnostic biomarker through online analysis of genomic and expression data from glioma cohort studies. GlioMarker provides a powerful, practical, and user-friendly web-based tool that may serve as a specialized platform for clinicians and researchers by providing rapid and comprehensive knowledge of glioma diagnostic biomarkers to subsequently facilitates high-quality research and applications.

## Highlights

1. GlioMarker is the first comprehensive database for knowledge exploration of glioma diagnostic biomarkers. It may function as a professional platform for clinicians and researchers to promote high-quality research and the application of diagnostic biomarkers in glioma.
2. In GlioMarker, the original data of the diagnostic biomarkers were manually curated based on full-text review, and ultimately each article was consolidated around the 33 fields we covered. From a literature perspective, the accurate and comprehensive coverage of curated information provides evidence for the potential clinical diagnostic biomarkers.
3. We integrated many external resources to provide clinicians and researchers with the ability to explore ontology, precision medical knowledge, and genomic and expression data of glioma patients for a better understanding of the biological significance of the diagnostic biomarkers.

## Introduction

Gliomas, arising from the glial support cells within the brain, are classified into four grades (World Health Organization [WHO] grades I-IV), and are the most common primary tumors of the central nervous system (CNS) (1). Histologically, gliomas include ependymomas, astrocytoma (including glioblastoma [GBM]), oligodendroglioma, mixed gliomas, and a few others, such as optic nerve and brain stem gliomas (2), exhibiting a considerable variability in age of onset, grade of severity, and ability to progress, as well as to metastasize (3). Due to the absence of effective diagnostic strategies, patients mainly rely on neurological examination and neuroimaging methods performed when the disease is already at an advanced stage, especially in glioblastoma (4). Therefore, early and efficient diagnosis is essential for implementing of precise therapy, which is crucial for prolonging patients’ lives and improving their quality of life (5).

Effective medical practice is clinically dependent on high-fidelity and technically suitable diagnostic biomarkers to accurately diagnose diseases and conditions (6). In recent decades, many researchers have focused on the study of new diagnostic biomarkers and their functions in gliomas (7, 8). In landmark 2016, the WHO classification of gliomas used molecular parameters in addition to histology to define many tumor entities for the first time, significantly improving the accuracy of tumor diagnosis (9). Thus, a thorough and comprehensive understanding of the existing diagnostic biomarkers in gliomas and the identification of more valuable potential biomarkers are of great significance in the era of precision oncology (5).

Although outstanding achievements have been made in biomarker research of glioma diagnosis, many challenges still exist. (i) Due to the lack of systematic knowledge, the molecular mechanism of glioma pathogenesis, malignancy, and clinical aggressiveness remains unclear. (ii) With the rapid increase in publications, it becomes a complex, time-consuming, and challenging process for biologists or clinicians to mine crucial diagnostic biomarkers and obtain knowledge from the multifarious literature and data sources with corroborative analysis. (iii) With high-throughput sequencing and multi-omics technology, glioma molecular characteristics have gradually been understood (10). Therefore, integrating glioma-related omics data with biomarker information from the literature to mine their biological functions and regulatory mechanisms through online analysis is worthy of further exploration. (iv) Although several biomarker databases have been established, such as the Tuberculosis Biomarker Database for tuberculosis (11), LiverCancerMarkerRIF (12) and CancerLivER (13) for liver cancer, ExoBCD (14) for exosomal biomarkers in breast cancer, and CBD (15) for colorectal cancer, no diagnostic biomarker database in gliomas has been reported for public usage, which highlights the need for the research and clinical application of biomarkers for glioma diagnosis.

This study developed an integrated and web-based database GlioMarker (http://gliomarker.prophetdb.org/) to overcome the above limitation. GlioMarker is part of the Prophet project, which aims to achieve the goal of establishing a cancer knowledge map ecosystem. In the current GlioMarker (version 1.0), accurate information on 406 glioma diagnostic biomarkers from 1559 publications (1989.05-2022.05), including biomarker descriptions, clinical information, associated literature, experimental records, associated diseases, statistical indicators, etc., was manually extracted. To better understand the biological significance of these biomarkers, we integrated many external resources to provide clinicians and researchers with capabilities for exploring ontology, precision medical knowledge, and genomic and expression data of glioma patients. GlioMarker can provide rapid and comprehensive knowledge of glioma diagnostic biomarkers for clinicians and researchers, facilitating high-quality research and applications as a powerful and practical tool with a user-friendly interface. In the future, multiple types of biomarkers will be covered in GlioMarker version 2.0.

## Materials and methods

**Figure 1:**
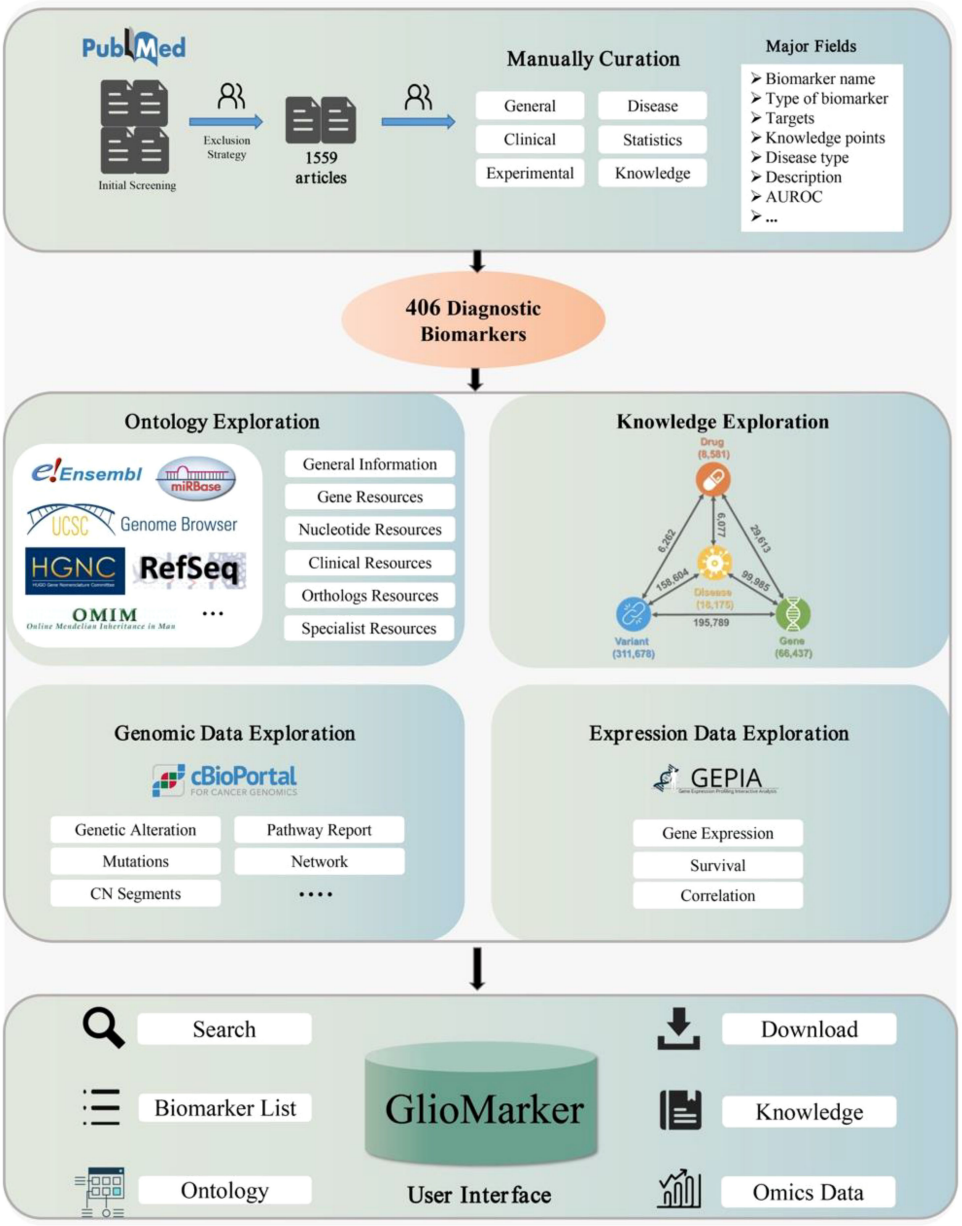
The flow chart of GlioMarker construction.

The flowchart of GlioMarker construction is shown in **Figure 1**, including literature-based mining and the establishment of biomarker knowledge exploration. The second part includes ontology annotations, precision medical knowledge exploration, and genomic and expression data exploration. The details are described in the following sections.

### Literature survey and data selection criteria

**Figure 2:**
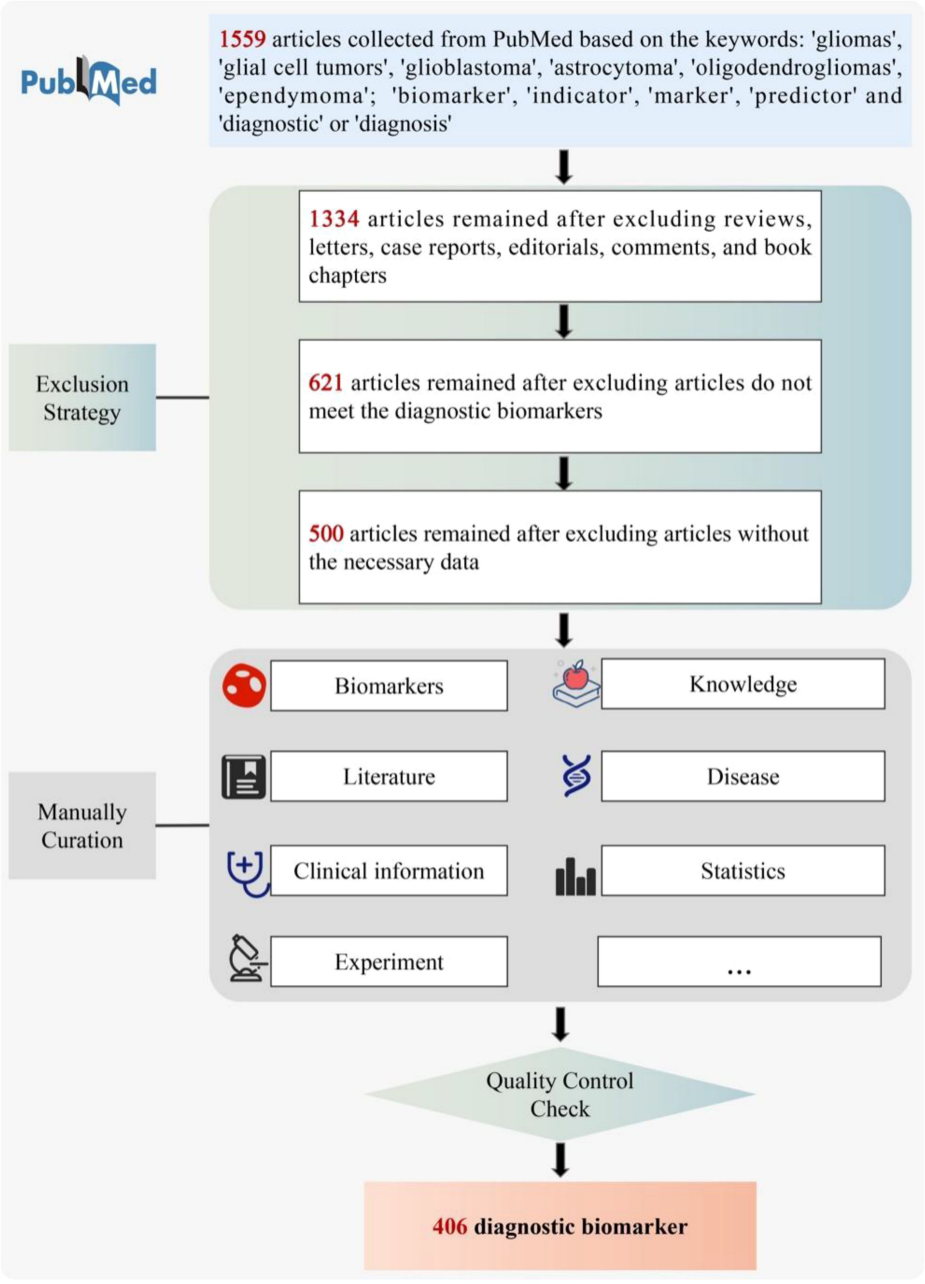
The pipeline of literature curation.

To guarantee high-quality of data collection, all the data for GlioMarker were collected from the public database PubMed (https://pubmed.ncbi.nlm.nih.gov/) by manual text mining as follows (**Figure 2**). First, a the detailed literature search was performed in PubMed using the following keywords: ((Biomarker[Title/Abstract]) OR (marker[Title/Abstract]) OR (indicator[Title/Abstract]) OR (predictor[Title/Abstract])) AND ((Glioma[Title/Abstract]) OR (Glial Cell Tumors[Title/Abstract]) OR(glioblastoma[Title/Abstract]) OR (astrocytoma[Title/Abstract]) OR (oligodendroglioma[Title/Abstract])) AND ((Diagnosis[Title/Abstract]) OR (Examinations[Title/Abstract]) OR (Diagnostic[Title/Abstract])). Based on these criteria, we collected 1559 articles (published 1989.05-2022.05) from PubMed as the original data.

We then filtered these articles according to the following specifications: (1) Only full publications were considered to collect, whereas case reports, communication letters, comments, and review articles were excluded (1334 were retained). (2) After reading the titles and abstracts, articles that did not meet the diagnostic biomarkers relationship were excluded (621 retained). (3) Articles without the necessary data were excluded (500 retained).

### Information extraction

We curated the 500 articles manually by reading the full-text, and two independent curators reviewed each returned article. Their curated information was then integrated. Other members of our team routinely performed additional quality checks.

Information about the diagnostic biomarkers was adequately extracted based on 33 fields, covering biomarker descriptions, clinical information, associated literature, experimental records, associated diseases, statistical indicators, etc. Importantly, we manually sorted out the knowledge points of each biomarker, which were defined as the original results of the biomarker. The data dictionary for the 33 fields is presented in **Supplementary Table S1**.

It is worth noting that if a biomarker was published several times, multiple corresponding records were included in GlioMarker. After quality control checks, 406 diagnostic biomarker entries were finally included in GlioMarker.

### The establishment of biomarker knowledge exploration

We integrated many external resources to provide GlioMarker with further knowledge exploration functions of diagnostic biomarkers obtained from the literature mining.

(1) Ontology exploration. In this function, users can obtain more ontology information about the biomarker. Ontology annotations were supported by HUGO (16), NCBI Gene (17), Ensembl BioMart (18), UCSC (19), RefSeq (20), RNAcentral (21), OMIM (22), miRBase (23), RGD (24), MGI (25), lncRNAdb (26), and LNCipedia (27).
(2) Precision medical knowledge exploration. In this function, users can find the relationship between any two or more components of diseases, drugs, genes, and variants to explore the knowledge of precision medicine, which was supported by PreMedKB (28).
(3) Omics data exploration. With this function, users can explore the clinical application value of a specific diagnostic biomarker through online analysis of genomic and expression data from glioma cohort studies. The visualization of genomic and expression data was based on the customized cBioPortal (29) and GEPIA (30) platforms, respectively.

### Database construction

GlioMarker was constructed in PostgreSQL (10.0), Django, and Python (3.8). HTML, CSS, JavaScript, and Vuejs were used to build the web interface. The Nginx was selected as the HTTP Server. These web operations were implemented in the CentOS (7.5.1804) operating system.

## Results

### GlioMarker statistics

In summary, a total of 406 diagnostic biomarkers were collected in GlioMarker. Based on the clinical use, these diagnostic biomarkers can also be used for prognosis, treatment, and prediction. Based on the biomolecule type, the 406 biomarkers can be classified into 7 categories, including protein (n=192), RNA (n=120), DNA (n=31), imageological (n=26), epigenetic (n=14), metabolic (n=12), and immunological (n=11) biomarkers. Among the 120 RNA biomarkers, 74 miRNAs, 27 lncRNAs, 11 mRNAs, 7 circRNAs, and 1 piRNA were included. Here, these biomarkers were discovered from major sources, such as tissue(n=262), serum(n=49), image(n=25), cell lines(n=18), plasma(n=17), cerebrospinal fluid (CSF, n=9), and urine(n=2). The detection methods of the biomarkers involve qRT-PCR, Western blotting, immunohistochemistry, ELISA, NMR, etc. Moreover, these diagnostic biomarker studies were performed in 39 different countries and focused on different subtypes, including glioma-with-no-classification (n=255, 62.8%), glioblastoma (n=113, 27.8%), other astroglioma (n=27, 6.7%), oligodendroglioma (n=7, 1.7%), mixed glioma (oligoastrocytoma) (n=3, 0.7%) and ependymoma (n=1, 0.2%). Statistics of the biomarker distribution in GlioMarker are provided in **Table 1**.

**Table 1:** Statistics of the distribution of biomarkers in GlioMarker.

Based on clinical uses		Based on biomolecules	
Diagnositic	128	Protein Biomarker	192
Diagnositic, Prognostic	128	miRNA	74
Diagnositic, Therapeutic	90	lncRNA	27
Diagnositic, Prognostic, Therapeutic	52	mRNA	11
Diagnositic, Therapeutic, Predictive	4	circRNA	7
Diagnositic, Predictive	3	piRNA	1
Diagnositic, Prognostic, Predictive	1	DNA Biomarker	31
		Imageological Biomarker	26
		Epigenic Biomarker	14
		Immunological Biomarker	12
		Metabolic Biomarker	11
**Based on sources of samples**		**Based on disease classification**	
Tissue	262	Glioma	255
Serum	49	Glioblastoma/Astrocytoma	113
Image	25	Astrocytoma	27
Cell lines	18	Oligodendroglioma	7
Plasma	17	Mixed glioma (Oligoastrocytoma)	3
Tissue, Serum	11	Ependymoma	1
Peripheral blood	10		
Cerebrospinal fluid (CSF)	9		
Serum, Cerebrospinal fluid (CSF)	2		
Urine	2		
Plasma, Urine	1		

The receiver operating characteristic curve (ROC), its associated area (AUC_ROC_), sensitivity, and specificity, are essential to globally assess the diagnostic performance of a biomarker in clinical use (31). Thus, in the process of literature extraction, these relevant indicators were included in GlioMarker. Among them, 81 biomarkers have been evaluated by ROC, and the AUC values were all greater than 0.6.

Importantly, we have assigned evidence levels to the biomarkers in the GlioMaker database. A total of 4 levels of evidence were defined, namely “Biomarker has been approved by the FDA”, “Biomarker is validated in clinical trials”, “Biomarker is validated in preclinical research (*in vitro* or *in vivo* models)”, and “Biomarker is putative one based on data analysis”, to indicate the robustness of the biomarkers. For each study, we manually compiled relevant knowledge about the biomarker, including its up regulators, targets, and knowledge points. In total, 1718 knowledge points, 16 up regulators, and 84 targets were retrieved.

Some diagnostic biomarkers that were reported multiple times may have more application prospects, and multiple corresponding records are provided in GlioMarker. Four different biomarker types are involved, among which GFAP (32–38) and miR-21 (39–45) were reported in more than 7 different publications (**Table 2**).

**Table 2:** List of genes/proteins reported as biomarkers/signatures in at least three different studies.

Gene symbol	Type of biomarker	Publications
GFAP	Protein Biomarker	7
MIR21	RNA Biomarker	7
IDH1	DNA Biomarker	5
MGMT	Epigenic Biomarker	4
MIR210	RNA Biomarker	4
OLIG2	Protein Biomarker	4
IDH2	DNA Biomarker	3
MAP2	Protein Biomarker	3
MIR124	RNA Biomarker	3
MIR15B	RNA Biomarker	3
MIR181B1	RNA Biomarker	3
MIR221	RNA Biomarker	3
PRKCA	DNA Biomarker	3
S100A8	Protein Biomarker	3

### Web interface

To allow users to better explore the glioma diagnostic biomarkers, an integrated GlioMarker with a user-friendly web interface was constructed. Database navigation occurs based on a set of menus, including HOME, BIOMARKER, DOWNLOAD, CURATION, ABOUT, and FEEDBACK.

**Figure 3:**
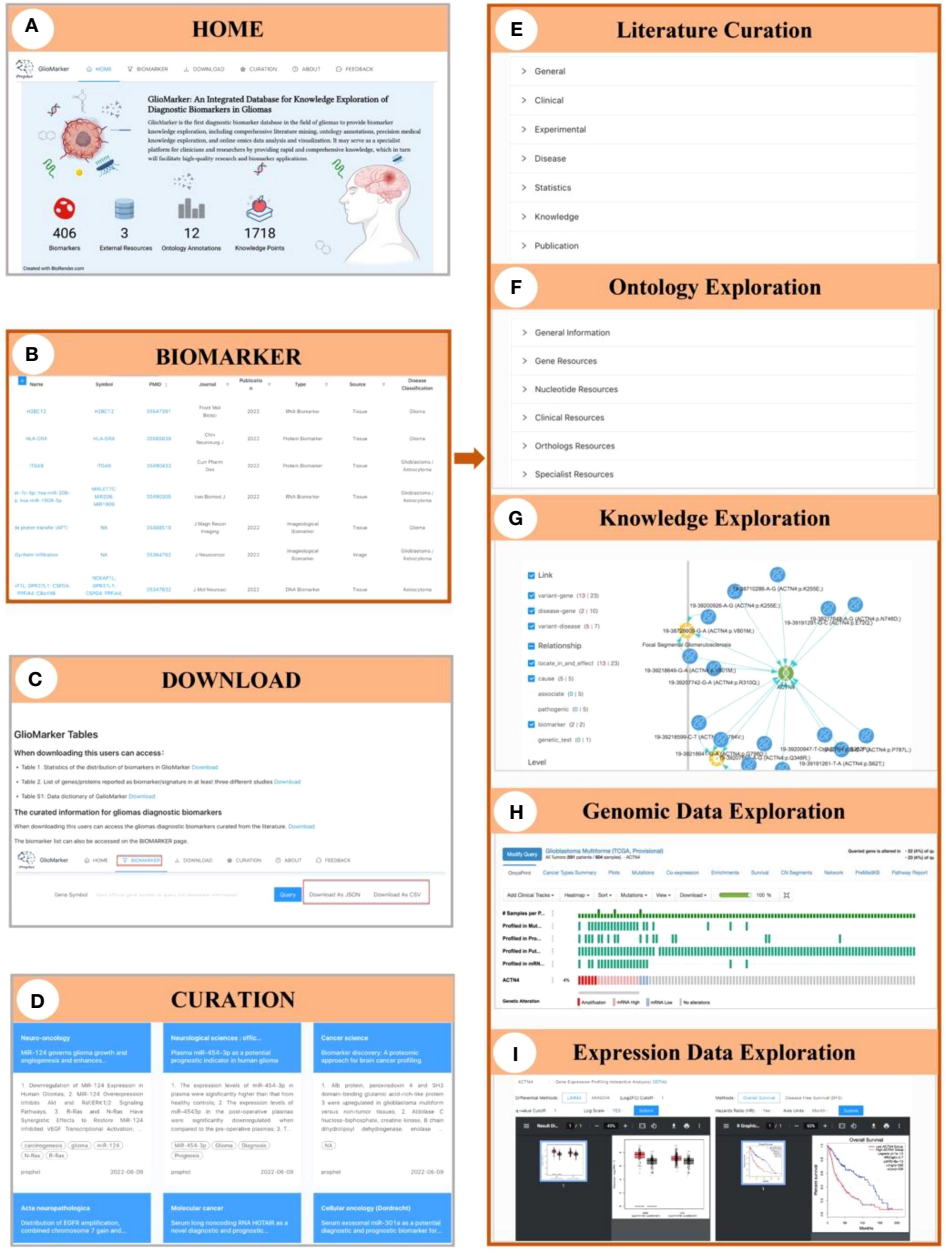
Illustration of the interface of GlioMarker. **(A)** Home page: six navigation menus and functional pages (HOME, BIOMARKER, DOWNLOAD, CURATION, ABOUT, and FEEDBACK. **(B)** BIOMARKER page: the diagnostic biomarkers list are provided. **(C)** DOWNLOAD page: the biomarker list and related documentation are available. **(D)** CURATION page: main content of the diagnositic biomarkers related publications were summarized. The details of biomarker knowledge exploration: literature curation **(E)**, ontology exprolation **(F)**, precision medical knowledge exploration **(G)**, and omics data online analysis **(H, I)**.

A navigation menu and a data summary block are presented on the HOME page (**Figure 3A**). Information on 406 biomarkers, 1718 knowledge points, 12 ontology annotations, and 3 integrated external resources is displayed in the data summary block.

On the BIOMARKER page, the diagnostic biomarkers list is provided (**Figure 3B**). The biomarker list has a filter function, and tabular information can be customized to allow users to build specific search strategies to suit different study designs. Keyword search accepts different types of names and accession IDs as search queries. Moreover, five subpages can be accessed by clicking on the name of a biomarker to facilitate further knowledge exploration: Curation, Ontology, Knowledge, Genomic Data, and Expression Data, respectively. On the Curation subpage, biomarker information extracted from the literature was displayed in detail and classified as “General”, “Clinical”, “Experimental”, “Disease”, “Statistics”, etc (**Figure 3E**). The Ontology subpage allows users to obtain more ontology annotations about the biomarker from other resources (**Figure 3F**). The Knowledge subpage displayed precision medicine exploration results of the relationship between diseases, drugs, genes, and variants (**Figure 3G**). The Genomic Data and Expression Data subpages provided online analysis of genomic and expression data from the glioma cohort study to explore the clinical application value of a specific diagnostic biomarker (**Figures 3H, I**).

On the CURATION page, the main contents of the diagnostic biomarker-related publications are summarized. The original articles are linked to PubMed *via* PMID (**Figure 3D**).

The biomarker list and related documentation are available on the DOWNLOAD page (**Figure 3C**). Help documents and our contact information are available on the ABOUT page. Finally, on the FEEDBACK page, users can provide comments or suggestions about GlioMarker.

### Case study

**Figure 4:**
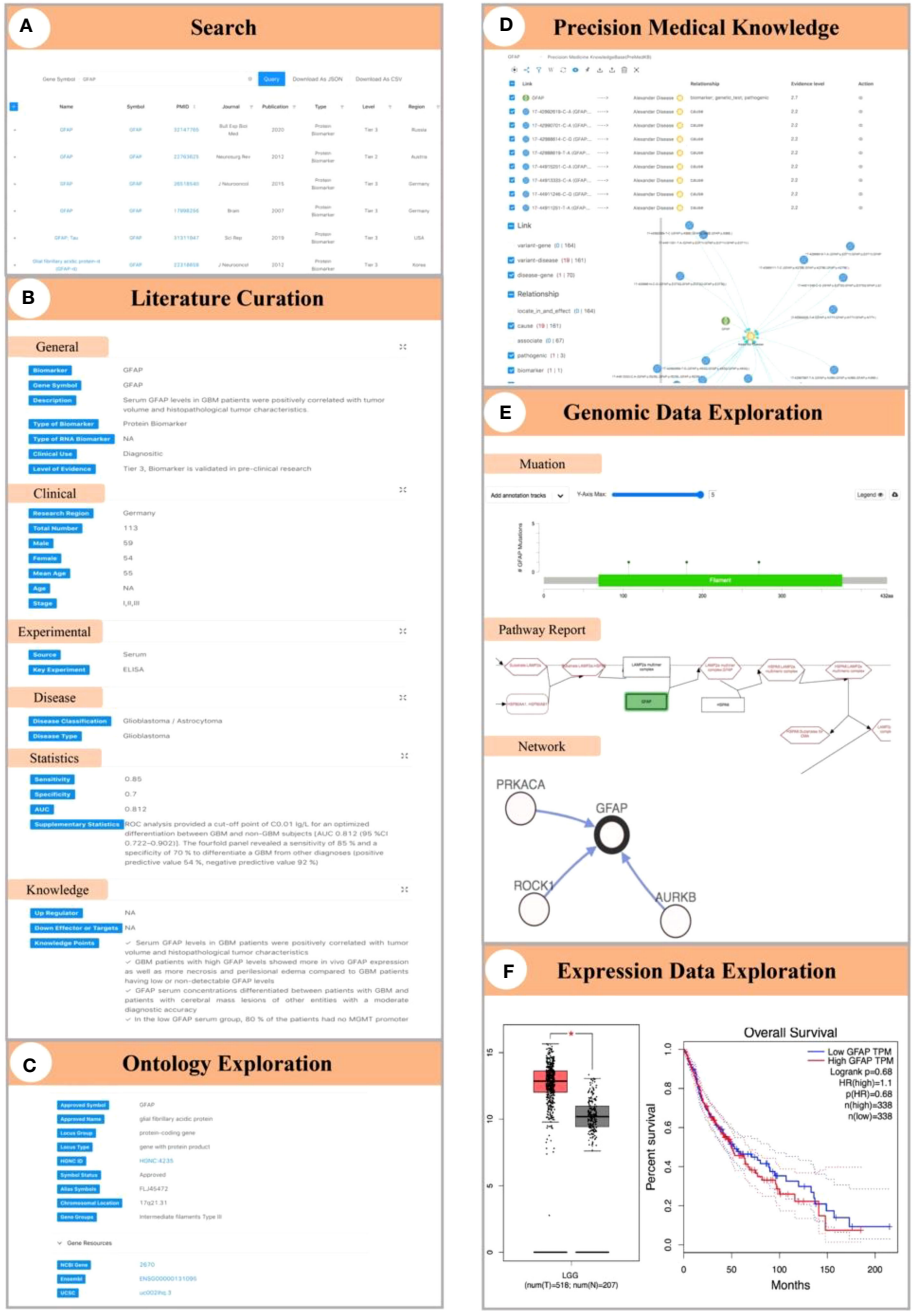
Demonstration of biomarker knowledge exploration in GlioMarker using GFAP as a case. **(A)** Seven studies on GFAP can be obtained by enter “GFAP” in the search box. **(B)** The results of literature curation on GFAP. **(C)** The ontology annotations of GFAP. **(D)** Precision medical knowledge exploration results on GFAP. **(E)** Explore the results of GFAP in genomic data (ie mutations, networks, pathway reports). **(F)** Explore the results of GFAP in the expression profile data.

Here, we first use GFAP as a case to illustrate biomarker knowledge exploration in GlioMarker. When the user entered “GFAP” in the search box, seven records of GFAP studies were available from 2007 to 2021 (**Figure 4A**). The results revealed that GFAP was significantly elevated in the plasma (33, 36) and serum (34, 35, 37) in glioblastoma (GBM) patients. Serum GFAP levels were able to distinguish GBM from non-GBM patients, and the maximum AUC was 0.9 (34). In addition, the expression pattern of GFAP-δ can also be used as a histopathological diagnostic biomarker for spinal astrocytoma (32) (**Figure 4B**). Users can further explore more knowledge of GFAP through the following functions. (1) In ontology exploration, users can access other external resources by using the hyperlink to obtain more ontology annotations (**Figure 4C**). (2) Precision medical knowledge exploration results showed many variants in GFAP, and some were significantly related to Alexander disease (**Figure 4D**). (3) Genomic data exploration revealed GFAP copy number alterations, networks, pathway reports, etc., at the genome level (**Figure 4E**). Among them, pathway reports indicate that GFAP is involved in autophagy signal transduction mediated by molecular chaperones, which may provide insights for further research on the mechanism of GFAP in the occurrence and development of gliomas. Moreover, the exploration of expression data shows that GFAP is highly expressed in low-grade glioma (LGG) patients, and this high expression is associated with a lower survival rate (**Figure 4F**).

In addition, GlioMarker has incorporated biomarkers that allow users to differentiate between low- or high-grade diseases. For example, CHI3L1(Alias symbols: YKL-40) was highly differentially expressed in high-grade glioma (HGG) tissue (46), and this protein can also be monitored in patients’ serum and help confirm the absence of active disease in GBM (47). Alterations in Galectin-1 (48), miR-766-5p and miR-376b-5p (49) levels in serum and ADLH1A1 (50), and WEE1 (51) levels in tissue might also be used as auxiliary diagnostic indicators of HGG. Moreover, users can also find potential LGG diagnostic biomarkers in GlioMarker. For example, the serum anti-FLNC autoantibody (52), the level of which was significantly higher in low-grade glioma patients than in high-grade glioma patients or in normal volunteers, represents a potential serum biomarker for the early diagnosis of LGG. In addition, HLA-DRA (53) and Fam20C (54) are also promising biomarkers for LGG diagnosis and prognosis.

GlioMarker can also aid in the identification of biomarkers suitable for liquid biopsies. By filtering “Source” in the columns and checking “Serum” and “Plasma”, 66 relevant studies were identified, and most of these studied forcus on circulating miRNAs. Taking miR-210 as an example, its robust level can be detected both in exosome (55), serum (56) and plasma (57) to distinguish patients with glioma from healthy controls, and maybe a promising diagnostic and prognostic biomarker. Additionally, through precision medical knowledge exploration, we found that miR-210 was also related to other diseases, such as siderosis (58)and pediatric osteosarcoma (59).

## Discussion

### Strengths of the GlioMarker

We compared GlioMarker with other specialized, open-access molecular biomarker databases, including The Tuberculosis Biomarker Database (11), LiverCancerMarkerRIF (12), CBD (15), CancerLivER (13), ExoBCD (14), the Urinary Protein Biomarker Database (UPBD) (60), and the Therapeutic Response Biomarker Database (ResMarkerDB) (61), and the strengths of GlioMarker are summarized below.

#### A wide variety of biomarker types

GlioMarker focused on glioma diagnostic biomarkers, which include a diverse range of biomolecule types. Biomarkers described in GlioMarker include protein, miRNA, lncRNA, circRNA, mRNA, DNA, as well as imageological, epigenetic, immunological, and metabolic features.

#### Comprehensive coverage of curated information

The original data of the diagnostic biomarkers were manually curated based on the full text. Each entry into GlioMarker was reviewed by two independent curators, and each biomarker was defined in the context of associated literature, experimental records, disease type, and clinical relevance, among other indicators. Therefore, information about the biomarkers in GlioMarker is more accurate and comprehensive. Importantly, these knowledge points are the actual results of the biomarker and help users quickly access the biomarker’s research content, providing evidence for the potential clinical diagnostic biomarker from a contextual perspective. This feature represents a significant advantage that distinguishes GlioMarker from other databases and will help construct reliable knowledge graphs in the future. GlioMarker will be updated every 12 months.

#### Powerful biomarker knowledge exploration capabilities

By integrating many external resources, clinicians or researchers can further explore the knowledge of these diagnostic biomarkers from three aspects. (1) Obtain more ontology annotations. (2) Identify the relationship between any two or more components of diseases, drugs, genes, and variants to explore information regarding precision medicine. (3) Explore the clinical application value of a specific diagnostic biomarker through online analysis of genomic and expression data from glioma cohort studies.

#### User-friendly search methods

In GlioMarker, users can access the biomarker of interest through the list or keyword search. The biomarker list has a filter function, allowing users to build specific search strategies to suit different study designs. The keyword search feature accepts different types of names and accession IDs as search queries.

#### Direct data transfer and integration.

GlioMarker allows users to extract knowledge they are interested in through convenient downloads. Users can also submit biomarker curation data created by themselves according to our template to integrate into GlioMarker.

### Limitations and future perspectives

According to the visions of predictive, preventive, personalized, and participatory medicine (P4 Medicine) (62–64), GlioMarker is a database focused on diagnostic biomarkers in glioma. While considerable effort has been made by our curation team to capture all the relevant information, there is no doubt that some biomarkers or newly emerging molecular biomarker types might have been missed. Similarly, although some diagnostic biomarkers included also have prognostic, therapeutic, or predictive values, GlioMarker currently does not include a sufficient number of prognostic, predictive, therapeutic, adverse drug effect, or drug efficacy biomarkers. We are actively exploring this area and expect that these biomarkers will be included in the 2.0 version of the database. In addition, the literature related to biomarkers is emerging in large numbers, so we need to filter the literature more strictly before curating in the next version, for example, by focusing on higher-quality papers and some rich biomarker performance data (i.e., quantitative expression results, sensitivity, specificity, ROC curve, reproducibility, statistical significance, threshold or cutoff value).

The 2016 WHO classification of central nervous tumors added molecular features to the histological diagnosis for the first time (9). With the rapid development of molecular oncology and the discovery of promising biological biomarkers, the 2021 edition adds more types/subtypes of tumors defined by biological and molecular characteristics and no longer reflects histological subtypes (65). We are inspired to witness these changes, and this is where GlioMarker’s vision lies. However, there is still some controversy regarding the 2021 edition classification, and in-depth follow-up studies are still needed. Many researchers believe that histologic morphology is still the primary requirement for diagnosing diffuse glioma and dose not rely exclusively on molecular alterations. For example, diffuse astrocytomas with lower histologic grade and IDH wild type with TERT promoter mutation are classified as glioblastoma in the 2021 fifth edition classification. However, different histologic grades still have different prognoses (66). In addition, since the new classification system was newly proposed, this change was not reflected in many previous studies. Therefore, the 2016 edition was still referenced in GlioMarker, and the classification of diseases according to histological morphology was retained. To improve the robustness and adaptability of GlioMarker, we plan to take the following measures to introduce new classification systems in subsequent versions. For example, by adding new fields to label the types of diseases classified according to the 2021 fifth edition, using typing trees can be used to help researchers adapt to the old and new editions of the classification, etc.

In the era of precision medicine, the application of biomarkers is crucial. However, many biomarkers remain in the stage of scientific research and are still far away from clinical application. Our Prophet project is committed to building a broad tumor biomarker platform and becoming a bridge connecting scientific research and clinical applications. GlioMarker aims to create a comprehensive biomarker platform in glioma and become a bridge between scientific research and clinical practice.

In addition, most of the biomarkers contained in GlioMarker only have potential application value, and the underlying mechanisms for most diagnostic biomarkers remain unknown. Therefore, in the next version of GlioMarker, we will establish a knowledge map based on knowledge points, and more omics data will be integrated. The biomarkers in GlioMarker will also be used for further meta-analysis to obtain more robust evidence. We are looking forward to more participation in the user community and receiving feedback on GlioMarker version 1.0.

## Conclusion

GlioMarker is the first diagnostic biomarker database in the field of gliomas to provide biomarker knowledge exploration, including comprehensive literature mining, ontology annotations, precision medical knowledge exploration, and online omics data analysis and visualization. GlioMarker may function as a professional platform for clinicians and researchers to promote the high-quality research and application of diagnostic biomarkers in gliomas as a powerful and practical web-based tool with a user-friendly interface.

## Data availability statement

The datasets presented in this study can be found in online repositories. The names of the repository/repositories and accession number(s) can be found in the article/**Supplementary Material**.

## Author contributions

ZR, JY, and YL, the main author of the study, conceived the study and contributed to the writing. ZR, JY, YL, XL, and SS took part in designing and conducting the study. XC, ZM, SW, XL, SS and ZR manually collected literature, YG, SZ, MF, JL, and ZR curated the biomarkers-related data. JY, YS, and YH developed the web interface. QC and ZC helped in the interpretation and analysis of data. TY contributed to the manuscript review, revision, and funding support. All of the authors read and approved the final manuscript.

## Funding

This work was funded by Shanghai Sailing Program (21YF1418700), Shanghai Municipal Education Commission (ZZSHJKYXY20008), Shanghai Pudong New Area Health Care Committee General Program (PW2019A-26), and Shanghai University of Medicine & Health Sciences (SSF-21-05-008, A3-0200-21-311007-33 and CFDQ20220004).

## Acknowledgments

The authors would like to thank The Genius Medicine Consortium (TGMC) for providing the computer cluster.

## Conflict of interest

The authors declare that the research was conducted in the absence of any commercial or financial relationships that could be construed as a potential conflict of interest.

## Publisher’s note

All claims expressed in this article are solely those of the authors and do not necessarily represent those of their affiliated organizations, or those of the publisher, the editors and the reviewers. Any product that may be evaluated in this article, or claim that may be made by its manufacturer, is not guaranteed or endorsed by the publisher.

## Supplementary material

Supplementary Table 1 The data dictionary.

Supplementary Table 2 The curated information of the diagnostic biomarkers.

## Abbreviations

WHO, World Health Organization; CNS, Central Nervous System; GBM, Glioblastoma CSF, Cerebrospinal Fluid; HGG, High-grade glioma; LGG, Low-grade glioma.

## References

[1] Omuro A DeAngelis LM. Glioblastoma and other malignant gliomas: A clinical review. JAMA (2013) 310:1842–50. doi: 10.1001/jama.2013.280319 24193082

[2] Ostrom QT Gittleman H Liao P Vecchione-Koval T Wolinsky Y Kruchko C. CBTRUS statistical report: Primary brain and other central nervous system tumors diagnosed in the united states in 2010–2014. Neuro Oncol (2017) 19:v1–v88. doi: 10.1093/neuonc/nox158 29117289 PMC5693142

[3] Davis ME. Epidemiology and overview of gliomas. Semin Oncol Nurs (2018) 34:420–9. doi: 10.1016/j.soncn.2018.10.001 30392758

[4] Silantyev AS Falzone L Libra M Gurina OI Kardashova KS Nikolouzakis TK. Current and future trends on diagnosis and prognosis of glioblastoma: From molecular biology to proteomics. Cells (2019) 8:863. doi: 10.3390/cells8080863 PMC6721640 31405017

[5] Jameson JL Longo DL. Precision medicine–personalized, problematic, and promising. N Engl J Med (2015) 372:2229–34. doi: 10.1056/NEJMsb1503104 26014593

[6] Vargas AJ Harris CC. Biomarker development in the precision medicine era: lung cancer as a case study. Nat Rev Cancer (2016) 16:525–37. doi: 10.1038/nrc.2016.56 PMC6662593 27388699

[7] Molinaro AM Taylor JW Wiencke JK Wrensch MR. Genetic and molecular epidemiology of adult diffuse glioma. Nat Rev Neurol (2019) 15:405–17. doi: 10.1038/s41582-019-0220-2 31227792 PMC7286557

[8] Ludwig K. Molecular markers in glioma. J Neurooncol (2017) 0:0–0. doi: 10.1007/s11060-017-2379-y PMC5568999 28233083

[9] Louis DN Perry A Reifenberger G Deimling A Figarella-Branger D Cavenee WK. The 2016 world health organization classification of tumors of the central nervous system: a summary. Acta Neuropathol (2016) 131:803–20. doi: 10.1007/s00401-016-1545-1 27157931

[10] Chen LH Pan C Diplas BH Xu C Hansen LJ Wu Y. The integrated genomic and epigenomic landscape of brainstem glioma. Nat Commun (2020) 11:3077–11. doi: 10.1038/s41467-020-16682-y PMC7299931 32555164

[11] Yerlikaya S Broger T MacLean E Pai M Denkinger CM. A tuberculosis biomarker database: the key to novel TB diagnostics. Int J Infect Dis (2017) 56:253–7. doi: 10.1016/j.ijid.2017.01.025 28159577

[12] Dai H-J. LiverCancerMarkerRIF: A liver cancer biomarker interactive curation system combining text mining and expert annotations. Database 2014. (2014). 10.1093/database/bau085 PMC4147259 25168057

[13] Kaur H Bhalla S Kaur D Raghava GP. CancerLivER: a database of liver cancer gene expression resources and biomarkers. Database (2020) 2020. doi: 10.1093/database/baaa012 PMC7061090 32147717

[14] Wang X Chai Z Pan G Hao Y Li B Ye T. ExoBCD: a comprehensive database for exosomal biomarker discovery in breast cancer. Brief Bioinform (2020) 68:394–14. doi: 10.1093/bib/bbaa088 32591816

[15] Zhang X Sun X-F Cao Y Ye B Peng Q Liu X. CBD: a biomarker database for colorectal cancer. Database (2018) 2018:2564–12. doi: 10.1093/database/bay046 PMC6007224 29846545

[16] Bruford EA Lush MJ Wright MW Sneddon TP Povey S Birney E. The HGNC database in 2008: a resource for the human genome. Nucleic Acids Res (2008) 36:D445–8. doi: 10.1093/nar/gkm881 PMC2238870 17984084

[17] Brown GR Hem V Katz KS Ovetsky M Wallin C Ermolaeva O. Gene: a gene-centered information resource at NCBI. Nucleic Acids Res (2015) 43:D36–42. doi: 10.1093/nar/gku1055 PMC4383897 25355515

[18] Kinsella RJ Kähäri A Haider S Zamora J Proctor G Spudich G. Ensembl BioMarts: a hub for data retrieval across taxonomic space. Database (2011) 2011:bar030. doi: 10.1093/database/bar030 21785142 PMC3170168

[19] Navarro Gonzalez J Zweig AS Speir ML Schmelter D Rosenbloom KR Raney BJ. The UCSC genome browser database: 2021 update. Nucleic Acids Res (2021) 49:D1046–57. doi: 10.1093/nar/gkaa1070 PMC7779060 33221922

[20] O'Leary NA Wright MW Brister JR Ciufo S Haddad D McVeigh R. Reference sequence (RefSeq) database at NCBI: current status, taxonomic expansion, and functional annotation. Nucleic Acids Res (2016) 44:D733–45. doi: 10.1093/nar/gkv1189 PMC4702849 26553804

[21] The RNAcentral consortium. RNAcentral: a hub of information for non-coding RNA sequences. Nucleic Acids Res (2019) 47:D221–9. doi: 10.1093/nar/gky1034 PMC6324050 30395267

[22] Amberger JS Bocchini CA Schiettecatte F Scott AF Hamosh A. OMIM.org: Online mendelian inheritance in man (OMIM®), an online catalog of human genes and genetic disorders. Nucleic Acids Res (2015) 43:D789–98. doi: 10.1093/nar/gku1205 PMC4383985 25428349

[23] Griffiths-Jones S Grocock RJ van Dongen S Bateman A Enright AJ. miRBase: microRNA sequences, targets and gene nomenclature. Nucleic Acids Res (2006) 34:D140–4. doi: 10.1093/nar/gkj112 PMC1347474 16381832

[24] Smith JR Hayman GT Wang S-J Laulederkind SJF Hoffman MJ Kaldunski ML. The year of the rat: The rat genome database at 20: a multi-species knowledgebase and analysis platform. Nucleic Acids Res (2020) 48:D731–42. doi: 10.1093/nar/gkz1041 PMC7145519 31713623

[25] Eppig JT. Mouse genome informatics (MGI) resource: Genetic, genomic, and biological knowledgebase for the laboratory mouse. ILAR J (2017) 58:17–41. doi: 10.1093/ilar/ilx013 28838066 PMC5886341

[26] Amaral PP Clark MB Gascoigne DK Dinger ME Mattick JS. lncRNAdb: a reference database for long noncoding RNAs. Nucleic Acids Res (2011) 39:D146–51. doi: 10.1093/nar/gkq1138 PMC3013714 21112873

[27] Volders P-J Helsens K Wang X Menten B Martens L Gevaert K. LNCipedia: a database for annotated human lncRNA transcript sequences and structures. Nucleic Acids Res (2013) 41:D246–51. doi: 10.1093/nar/gks915 PMC3531107 23042674

[28] Yu Y Wang Y Xia Z Zhang X Jin K Yang J. PreMedKB: an integrated precision medicine knowledgebase for interpreting relationships between diseases, genes, variants and drugs. Nucleic Acids Res (2019) 47:D1090–101. doi: 10.1093/nar/gky1042 PMC6324052 30407536

[29] Cerami E Gao J Dogrusoz U Gross BE Sumer SO Aksoy BA. The cBio cancer genomics portal: an open platform for exploring multidimensional cancer genomics data. Cancer Discov (2012) 2:401–4. doi: 10.1158/2159-8290.CD-12-0095 PMC3956037 22588877

[30] Tang Z Li C Kang B Gao G Li C Zhang Z. GEPIA: a web server for cancer and normal gene expression profiling and interactive analyses. Nucleic Acids Res (2017) 45:W98–W102. doi: 10.1093/nar/gkx247 28407145 PMC5570223

[31] Ray P Le Manach Y Riou B Houle TT. Statistical evaluation of a biomarker. J Am Soc Anesthesiologists (2010) 112:1023–40. doi: 10.1097/ALN.0b013e3181d47604 20234303

[32] Heo DH Kim SH Yang K-M Cho YJ Kim KN Yoon DH. A histopathological diagnostic marker for human spinal astrocytoma: expression of glial fibrillary acidic protein-δ. J Neurooncol (2012) 108:45–52. doi: 10.1007/s11060-012-0801-z 22318658

[33] Lewis J Alattar AA Akers J Carter BS Heller M Chen CC. A pilot proof-Of-Principle analysis demonstrating dielectrophoresis (DEP) as a glioblastoma biomarker platform. Sci Rep (2019) 9:10279–10. doi: 10.1038/s41598-019-46311-8 PMC6635369 31311947

[34] Jung CS Foerch C Schänzer A Heck A Plate KH Seifert V. Serum GFAP is a diagnostic marker for glioblastoma multiforme. Brain (2007) 130:3336–41. doi: 10.1093/brain/awm263 17998256

[35] Tichy J Spechtmeyer S Mittelbronn M Hattingen E Rieger J Senft C. Prospective evaluation of serum glial fibrillary acidic protein (GFAP) as a diagnostic marker for glioblastoma. J Neurooncol (2016) 126:361–9. doi: 10.1007/s11060-015-1978-8 26518540

[36] Ilhan-Mutlu A Wagner L Widhalm G Wöhrer A Bartsch S Czech T. Exploratory investigation of eight circulating plasma markers in brain tumor patients. Neurosurg Rev (2013) 36:45–55. doi: 10.1007/s10143-012-0401-6 22763625

[37] Lyubimova NV Timofeev YS Mitrofanov AA Bekyashev AK Goncharova ZA Kushlinskii NE. Glial fibrillary acidic protein in the diagnosis and prognosis of malignant glial tumors. Bull Exp Biol Med (2020) 168:503–6. doi: 10.1007/s10517-020-04741-9 32147765

[38] van Bodegrave EJ Sluijs JA Tan AK Robe PAJT Hol EM. New GFAP splice isoform (GFAPµ) differentially expressed in glioma translates into 21 kDa n-terminal GFAP protein. new GFAP splice isoform (GFAPµ) differentially expressed in glioma translates into 21 kDa n-terminal GFAP protein. FASEB J (2021) 35:e21389. doi: 10.1096/fj.202001767R 33583081 PMC12266313

[39] Baraniskin A Kuhnhenn J Schlegel U Maghnouj A Zöllner H Schmiegel W. Identification of microRNAs in the cerebrospinal fluid as biomarker for the diagnosis of glioma. Neuro Oncol (2012) 14:29–33. doi: 10.1093/neuonc/nor169 21937590 PMC3245991

[40] Shi R Wang P-Y Li X-Y Chen J-X Li Y Zhang X-Z. Exosomal levels of miRNA-21 from cerebrospinal fluids associated with poor prognosis and tumor recurrence of glioma patients. Oncotarget (2015) 6:26971–81. doi: 10.18632/oncotarget.4699 PMC4694967 26284486

[41] Ivo D'Urso P Fernando D'Urso O Damiano Gianfreda C Mezzolla V Storelli C Marsigliante S. miR-15b and miR-21 as circulating biomarkers for diagnosis of glioma. Curr Genomics (2015) 16:304–11. doi: 10.2174/1389202916666150707155610 PMC4763968 27047250

[42] Koshkin PA Chistiakov DA Nikitin AG Konovalov AN Potapov AA Usachev DY. Analysis of expression of microRNAs and genes involved in the control of key signaling mechanisms that support or inhibit development of brain tumors of different grades. Clin Chim Acta (2014) 430:55–62. doi: 10.1016/j.cca.2014.01.001 24412320

[43] Santangelo A Imbrucè P Gardenghi B Belli L Agushi R Tamanini A. A microRNA signature from serum exosomes of patients with glioma as complementary diagnostic biomarker. J Neurooncol (2018) 136:51–62. doi: 10.1007/s11060-017-2639-x 29076001

[44] Zhi F Chen X Wang S Xia X Shi Y Guan W. The use of hsa-miR-21, hsa-miR-181b and hsa-miR-106a as prognostic indicators of astrocytoma. Eur J Cancer (2010) 46:1640–9. doi: 10.1016/j.ejca.2010.02.003 20219352

[45] Nikolova E Georgiev C Laleva L Milev M Spiriev T Stoyanov S. Diagnostic, grading and prognostic role of a restricted miRNAs signature in primary and metastatic brain tumours. discussion on their therapeutic perspectives. Mol Genet Genomics (2022) 297:357–71. doi: 10.1007/s00438-021-01851-5 35064290

[46] Nutt CL Betensky RA Brower MA Batchelor TT Louis DN Stemmer-Rachamimov AO. YKL-40 is a differential diagnostic marker for histologic subtypes of high-grade gliomas. Clin Cancer Res (2005) 11:2258–64. doi: 10.1158/1078-0432.CCR-04-1601 15788675

[47] Hormigo A Gu B Karimi S Riedel E Panageas KS Edgar MA. YKL-40 and matrix metalloproteinase-9 as potential serum biomarkers for patients with high-grade gliomas. Clin Cancer Res (2006) 12:5698–704. doi: 10.1158/1078-0432.CCR-06-0181 17020973

[48] Verschuere T Van Woensel M Fieuws S Lefranc F Mathieu V Kiss R. Altered galectin-1 serum levels in patients diagnosed with high-grade glioma. J Neurooncol (2013) 115:9–17. doi: 10.1007/s11060-013-1201-8 23824536

[49] Wang S Xu Z Zhang C Yu R Jiang J Wang C. High-throughput sequencing-based identification of serum exosomal differential miRNAs in high-grade glioma and intracranial lymphoma. BioMed Res Int (2020) 2020:2102645. doi: 10.1155/2020/2102645 33083454 PMC7563063

[50] Xu S-L Liu S Cui W Shi Y Liu Q Duan J-J. Aldehyde dehydrogenase 1A1 circumscribes high invasive glioma cells and predicts poor prognosis. Am J Cancer Res (2015) 5:1471–83. PMC4473324 26101711

[51] Shu C Wang Q Yan X Wang J. Whole-genome expression microarray combined with machine learning to identify prognostic biomarkers for high-grade glioma. J Mol Neurosci (2018) 64:491–500. doi: 10.1007/s12031-018-1049-7 29502292

[52] Adachi-Hayama M Adachi A Shinozaki N Matsutani T Hiwasa T Takiguchi M. Circulating anti-filamin c autoantibody as a potential serum biomarker for low-grade gliomas. BMC Cancer (2014) 14:452–10. doi: 10.1186/1471-2407-14-452 PMC4094678 24946857

[53] Chen D Yao J Hu B Kuang L Xu B Liu H. New biomarker: the gene HLA-DRA associated with low-grade glioma prognosis. Chin Neurosurg J (2022) 8:12–9. doi: 10.1186/s41016-022-00278-0 PMC9118678 35585639

[54] Feng J Zhou J Zhao L Wang X Ma D Xu B. Fam20C overexpression predicts poor outcomes and is a diagnostic biomarker in lower-grade glioma. Front Genet (2021) 12:757014. doi: 10.3389/fgene.2021.757014 34970298 PMC8712682

[55] Lan F Yue X Xia T. Exosomal microRNA-210 is a potentially non-invasive biomarker for the diagnosis and prognosis of glioma. Oncol Lett (2020) 19:1967–74. doi: 10.3892/ol.2020.11249 PMC7039075 32194691

[56] Lai N-S Wu D-G Fang X-G Lin Y-C Chen S-S Li Z-B. Serum microRNA-210 as a potential noninvasive biomarker for the diagnosis and prognosis of glioma. Br J Cancer (2015) 112:1241–6. doi: 10.1038/bjc.2015.91 PMC4385967 25756397

[57] Tabibkhooei A Izadpanahi M Arab A Zare-Mirzaei A Minaeian S Rostami A. Profiling of novel circulating microRNAs as a non-invasive biomarker in diagnosis and follow-up of high and low-grade gliomas. Clin Neurol Neurosurg (2020) 190:105652. doi: 10.1016/j.clineuro.2019.105652 31896490

[58] Lee D-C Romero R Kim J-S Tarca AL Montenegro D Pineles BL. miR-210 targets iron-sulfur cluster scaffold homologue in human trophoblast cell lines: siderosis of interstitial trophoblasts as a novel pathology of preterm preeclampsia and small-for-gestational-age pregnancies. Am J Pathol (2011) 179:590–602. doi: 10.1016/j.ajpath.2011.04.035 21801864 PMC3160082

[59] Cai H Lin L Cai H Tang M Wang Z. Prognostic evaluation of microRNA-210 expression in pediatric osteosarcoma. Med Oncol (2013) 30:499–6. doi: 10.1007/s12032-013-0499-6 23430441

[60] Shao C Li M Li X Wei L Zhu L Yang F. A tool for biomarker discovery in the urinary proteome: a manually curated human and animal urine protein biomarker database. Mol Cell Proteomics (2011) 10:M111.010975. doi: 10.1074/mcp.M111.010975 PMC3226410 21876203

[61] Pérez-Granado J Piñero J Furlong LI. ResMarkerDB: a database of biomarkers of response to antibody therapy in breast and colorectal cancer. Database (2019) 2019:12–1. doi: 10.1093/database/baz060 PMC6551372 31169290

[62] Hood L Friend SH. Predictive, personalized, preventive, participatory (P4) cancer medicine. Nat Rev Clin Oncol (2011) 8:184–7. doi: 10.1038/nrclinonc.2010.227. Nature Publishing Group. 21364692

[63] Hood L Flores M. A personal view on systems medicine and the emergence of proactive P4 medicine: predictive, preventive, personalized and participatory. N Biotechnol (2012) 29:613–24. doi: 10.1016/j.nbt.2012.03.004 22450380

[64] Cesario A Auffray C Russo P Hood L. P4 medicine needs P4 education. Curr Pharm Des (2014) 20:6071–2. doi: 10.2174/1381612820666140314145445 24641231

[65] Louis DN Perry A Wesseling P Brat DJ Cree IA Figarella-Branger D. The 2021 WHO classification of tumors of the central nervous system: a summary. Neuro Oncol (2021) 23:1231–51. doi: 10.1093/neuonc/noab106 PMC8328013 34185076

[66] Giannini C Giangaspero F. TERT promoter mutation: is it enough to call a WHO grade II astrocytoma IDH wild-type glioblastoma? Neuro Oncol (2021) 23:865–6. doi: 10.1093/neuonc/noab052 PMC8168806 33660766

